# Design and Analysis of a Passive Micromixer Based on Multiple Passages

**DOI:** 10.3390/mi16050592

**Published:** 2025-05-19

**Authors:** Makhsuda Juraeva, Dong-Jin Kang

**Affiliations:** School of Mechanical Engineering, Yeungnam University, 280 Daehak-ro, Gyoungsan 38541, Republic of Korea; mjuraeva@ynu.ac.kr

**Keywords:** multiple passages, degree of mixing (DOM), submergence, elongated interface, secondary flow

## Abstract

We propose a novel passive micromixer based on multiple passages and analyze its mixing performance comprehensively. The multiple passages are constructed with straight channels, making them easier to manufacture, compared to conventional SAR micromixers and other micromixers based on curved channels. Its mixing performance has been demonstrated to be superior to that of the previous micromixers across a broad range of Reynolds numbers. Five distinct designs incorporating converging passages were explored to study the significance of the number of passages on the mixing performance. Across a broad range of Reynolds number ranges (0.1 to 80), the two-passage design significantly improved mixing performance, with a degree of mixing (DOM) consistently exceeding 0.84. Particularly, the mixing enhancement is prominent within the low and intermediate range of Reynolds numbers (Re≤20). This enhancement in the regime of molecular diffusion dominance stems from the elongated interface between the two fluids. The mixing enhancement in the transition regime is due to a secondary flow being generated on the cross-section normal to the main stream direction. The intensity of this secondary flow is significantly influenced by the number of multiple passages. The optimal number for the present micromixer design is two. The DOM remains almost constant for the submergence of multiple passages in the range of 40 to 70 (μm).

## 1. Introduction

Microfluidic or lab-on-a-chip devices [1,2,3] have been of great interest over the decades. Although microfluidics technology emerged as early as the 1950s with the development of inkjet printers [4], significant advancements have been made in the past two decades. They are now widely applied in fields such as pharmaceutical technology, and biotechnology for chemical analysis, biomedical diagnostics, food and genetic engineering, drug delivery, and medicine. They serve multiple purposes, including delivering drugs and analyzing samples. Typically, they integrate functions such as injection, reaction, mixing, separation, and detection onto a chip. In this context, micromixers play a crucial role by homogenizing sample reagents at microscale, thus impacting the efficiency and sensitivity of the system, which are the key performance factors in microfluidic and lab-on-a-chip devices. Consequently, the design goals for micromixers include minimizing reagent consumption, maintaining a compact size, and achieving a faster mixing process [3,5].

Mixing in the microfluidic devices is usually achieved in the microchannels with excited turbulences and/or microstructures to increase the surface-to-volume ratio. Due to the microscale dimensions, resulting sluggish flow velocities lead to low Reynolds numbers, which can cause inefficient mixing. Therefore, designing an efficient micromixer is crucial to overcoming these inherent challenges. The advancement of the microfluidic or lab-on-a-chip industries heavily relies on the development of efficient micromixers. Although various efforts have been attempted to address these challenges, the field still requires novel mechanisms to achieve more efficient mixing [1,2,3,5].

Micromixer design focuses on enhancing mixing through either passive or active methods. Active micromixers typically require an additional energy source to agitate the fluid flow within the micromixer. Examples include acoustic micromixers [6], magneto-fluidic micromixers [7,8], electrothermal micromixers [9], photothermal micromixers [10], pulsating flow micromixers [11,12], or microscale electroosmosis [13]. While these extra energy sources offer the advantage of forcibly agitating flow, they also come with several drawbacks such as higher energy consumption and complex structure [2,7]. These limitations are particularly significant in the context of portability and cost-effectiveness [14].

In contrast, passive micromixers induce circulatory flows and increase the surface-to-volume ratio, solely through geometric complexity. This approach allows for more straightforward integration, making it cost-effective and adaptable within any microfluidic system. Examples of geometric complexity include split-and-recombine (SAR) [15,16,17], channel wall twisting [18], block in the junction [19], staggered herringbone [20], surface grooves and baffles [21,22], convergence-and–divergence [23], Tesla structure [24,25], mixing unit stacking [26,27], and submerged geometric structures [28,29,30].

Recent developments and research trends in the field of micromixers focus on three major areas: fabrication, material, and design [5]. A common objective is to develop a cost-effective micromixer. However, there is no universally superior technology for fabricating fluidic micromixers. The optimal choice depends on the device’s design and mixing performance [31]. To address this, micromixers should be designed to be as simple as possible while enhancing mixing performance. In this context, we propose a passive micromixer that utilizes straight and multiple passages to facilitate easy fabrication and improve mixing performance.

Biology and chemistry applications often demand rapid mixing times across a broad range of Reynolds numbers (Re < 100) [32,33,34,35,36]. In these applications, micromixing is primarily influenced by chaotic convection and molecular diffusion. Therefore, most passive micromixers incorporate various structures attached to their walls [15,16,17,18,19,20,21,22,23,24,25,26,27,28,29,30]. These geometric complexities have two contrasting effects on mixing performance. On one hand, they are generally expected to enhance molecular diffusion by elongating the interface between two fluids. On the other hand, they act as blockages in the fluid flow within a micromixer, leading to an increase in the required pressure drop. Therefore, designing effective passive micromixers requires a comprehensive understanding of how specific geometric complexities impact mixing performance across a broad range of Reynolds numbers. To the author’s knowledge, no research has yet been conducted to design and evaluate the mixing performance of a passive micromixer within this context.

In this paper, we present a novel design concept that seeks to balance the mixing mechanisms of molecular diffusion and chaotic convection. Our objective is to enhance mixing efficiency while minimizing flow blockages. To achieve this, we have chosen a geometric structure that increases the interface between the two fluids, arranged in a way that minimizes blockage effects on fluid flow. Specifically, we have constructed multiple passages using triangular structures to enhance the interface between the two fluids. The number of passages was carefully determined to minimize flow blockages, while also promoting flow acceleration through converging channel-like passages.

The micromixer introduced in this paper comprises six mixing units, each consisting of two chambers containing several triangular structures. These structures create multiple passages and facilitate the split-and-recombine mechanism of fluid flow, with flow acceleration along converging passages. The interface between two fluids is adjusted by the number of flow passages, which range from 0 to 4, to optimize their impact on mixing performance. The flow passage is constructed by straight channels to facilitate the manufacturing process. This design was simulated across a broad range of Reynolds numbers from 0.1 to 80. Its mixing performance was assessed by calculating the degree of mixing (DOM) at the outlet, along with the associated pressure drop.

## 2. Governing Equations and Computational Procedure

To evaluate the mixing performance of the present micromixer, simulations were conducted using ANSYS^®^ Fluent 2021 R2 [37]. The fluid flow and mixing performance was simulated by solving the 3D Navier–Stokes equation, the continuity equation, and a species convection–diffusion equation. Since the fluid flow is laminar, the governing equations are as follows:

(1) $$\left(\vec{u}\cdot \nabla \right)\vec{u}=-\frac{1}{\rho }\nabla p+\nu \nabla^{2}\vec{u}$$

(2) $$\nabla \cdot \vec{u}=0$$

where $\vec{u}$, *p*, and *ν* are the velocity vector, pressure, and kinematic viscosity, respectively. The evolution of mixing is simulated by solving a convection–diffusion equation:

(3) $$\left(\vec{u}\cdot \nabla \right)\varphi =D\nabla^{2}\varphi$$

where *D* and *φ* represent the mass diffusivity and mass fraction of a fluid, respectively.

The commercial software ANSYS^®^ FLUENT 2021 R2 [37], which employs the finite volume method, was used for the simulations. To discretize the convective terms in the governing Equations (1) and (3), the QUICK (quadratic upstream interpolation for convective kinematics) scheme was employed. This scheme is third-order accurate, providing more precise results for convective flows. In the simulation setup, fluid A and fluid B were introduced through inlet 1 and inlet 2, respectively. Both fluids were assumed to have properties similar to water, with a density of *ρ* = 997 kg/m^3^, a diffusion coefficient of *D* = 1.0 × 10^−10^ m^2^s^−1^, and a kinematic viscosity of *ν =* 0.97 × 10^−6^ m^2^s^−1^, respectively. The Schmidt (Sc) number for the fluids, which is a dimensionless number representing the ratio of momentum diffusivity (viscosity) to mass diffusivity, is approximately 10^4^. This high Sc number indicates that momentum diffuses much faster than mass. The Reynolds number Re, which characterizes the flow regime, was defined as $Re=\frac{\rho U_{mean}d_{h}}{\mu }$, based on the density ρ, the hydraulic diameter *d_h_*, the viscosity μ, and the mean velocity at the outlet $U_{mean}$.

The velocity distribution at both inlets, inlet 1 and inlet 2, was assumed to be uniform, while the outflow condition was applied at the outlet. The Knudsen number *Kn*, defined as the ratio of the mean free path length of fluid molecules to a characteristic length of the micromixer, was less than 10^−3^. Consequently, the no-slip boundary condition is applicable along all walls [38]. Fluid A was assumed to be supplied at the inlet 1. That is, the mass fraction of fluid A was *φ* = 1 at inlet 1 and *φ* = 0 at inlet 2.

The mixing performance was assessed in terms of DOM and MEC (mixing energy cost). DOM is defined as follows:

(4) $$DOM=1-\frac{1}{\xi }\sqrt{\sum_{i=1}^{n}\frac{{\left(\varphi_{i}-\xi \right)}^{2}}{n}},$$

where *φ_i_* and *n* are the mass fraction of fluid A in the *i*th cell and the total number of cells, respectively. *ξ* = 0.5 represents the state of complete mixing of two fluids. DOM = 1 means that the two fluids are completely mixed, while DOM = 0 indicates no mixing. MEC measures the effectiveness of present micromixer and is defined in the following form [39]:

(5) MEC=∆pρumean2DOM×100,

where $u_{mean}$ is the average velocity at the outlet, and ∆p is the pressure drop between the inlet and outlet.

## 3. Validation of the Numerical Study

For high Schmidt (Sc) number simulations, numerical diffusion can deteriorate the accuracy of simulated results. Several techniques have been proposed to minimize this numerical diffusion. One technique is to use a particle-based simulation methodology, such as the lattice Boltzmann equation [40] or the Monte Carlo method [41]. For grid-based numerical methods, reducing the cell Peclet number can be employed. For example, Bayareh [42] recommended restricting the cell Peclet number to $Pe_{c}\leq 2$. Here, the cell Peclet number $Pe_{c}=\frac{U_{cell}l_{cell}}{D}$ is based on flow velocity $U_{cell}$ and cell size $l_{cell}$. However, these approaches require substantial computational resources, making them impractical for studies like the present one. Instead of resorting to a computationally intensive approach, this paper employed a pragmatic approach, commonly adopted in many numerical studies [43].

**Figure 1:**
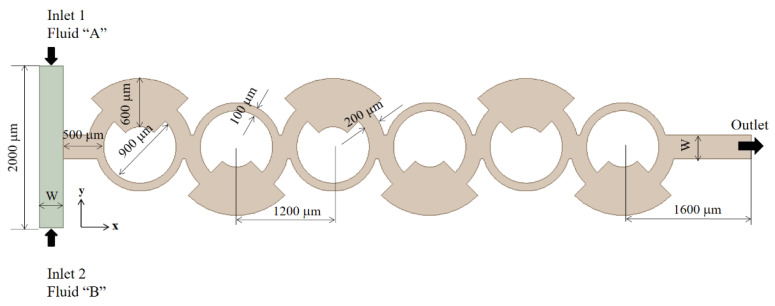
Diagram of the micromixer experimented by Xia et al. [44].

We validated the present numerical approach by simulating a passive micromixer experimented by Xia et al. [44]. This micromixer is illustrated schematically in Figure 1 and features a rectangular cross-section of W = 300 μm wide and 200 μm deep for both inlet channels. The micromixer consists of six mixing units, each containing a fan-shaped cavity, as illustrated in Figure 1. To ensure consistency with the experimental setup, the diffusion coefficient was set to 1.2 × 10^−9^ m^2^/s, the same value reported by Xia et al. [44]. Simulations were carried out for six different Reynolds numbers, specifically Re *=* 1, 10, 20, 40, 60 and 80. Following these simulations, a comprehensive comparison was made to demonstrate the accuracy and credibility of the present numerical approach.

We meshed the present micromixer shown in Figure 1 using a sufficient number of cells to ensure accurate numerical solutions. To determine the optimal mesh resolution, we conducted a set of preliminary simulations, varying the edge size from 5 μm to 7 μm. This resulted in cell numbers ranging from 4.43 × 10^6^ to 11.5 × 10^6^.

**Figure 2:**
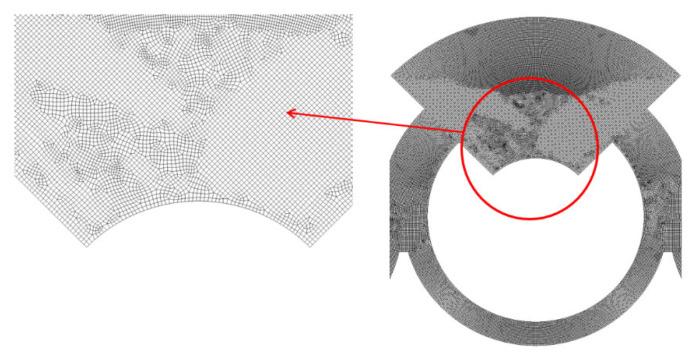
Grid within a mixing unit.

**Figure 3:**
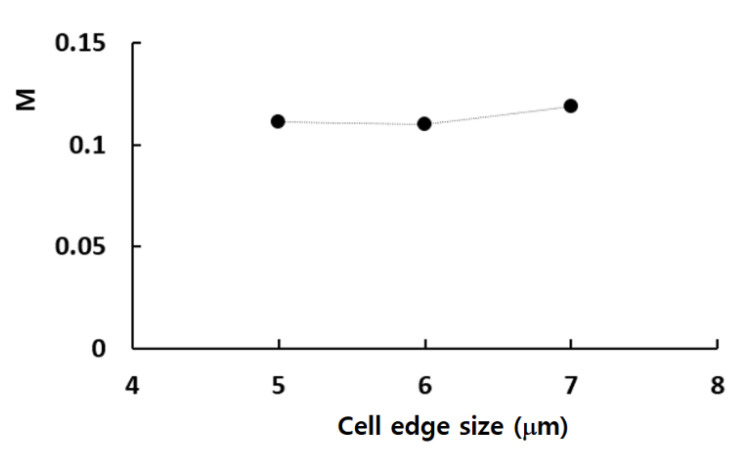
Grid independence of numerical solution.

For a more detailed examination, an enlarged view of the grid within a mixing unit is illustrated in Figure 2. According to Okuducu et al. [45], the type of cell used in the mesh can significantly impact the numerical accuracy. They recommended using hexahedral cells over prism and tetrahedral cells for better precision. As demonstrated in Figure 2, hexahedral cells were predominantly utilized to enhance the reliability of the numerical results. Figure 3 illustrates the grid dependence of the numerical solution with the edge size of cell. Here, *M* stands for the mixing index by Xia et al. [44], which is calculated in the following way:

(6) $$M=1-\frac{\sigma_{D}}{\sigma_{D,o}},$$

and

(7) $$\sigma =\sqrt{\frac{1}{n}\sum_{i=1}^{n}{\left(\varphi_{i}-\varphi_{opm}\right)}^{2}},$$

where $\sigma_{D}$ is the standard deviation of mass fraction φ, at any specific cross-section perpendicular to the flow direction, and $\sigma_{D,o}$ is the maximum value of $\sigma_{D}$ over the cross-section of the channel. n is the total number of sampling points at any cross-section. $\varphi_{opm}\;$ is the optimal value of φ, and it is 0.5. The simulation results demonstrate that the relative error of numerical solution is reduced to 0.87% at 6 μm from 7.8% at 7 μm. Consequently, all simulations for the grid independence test was carried out using an edge size of 6 μm, which corresponds to a cell number of 6.28 × 10^6^. This approach allowed us to balance computational efficiency with the accuracy needed for reliable results.

**Figure 4:**
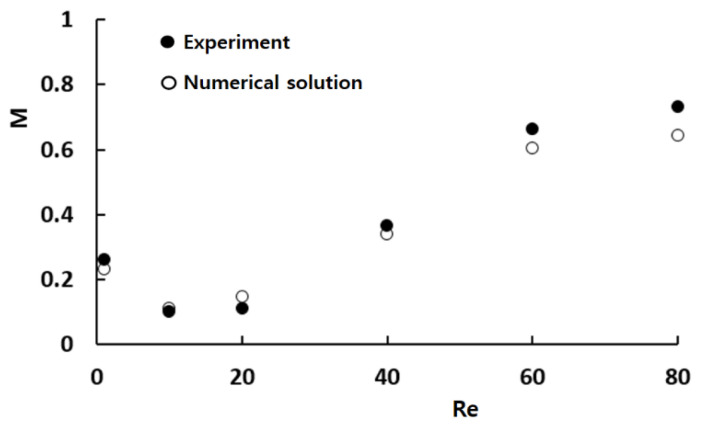
Comparison of numerical solutions with corresponding experimental data.

**Figure 5:**
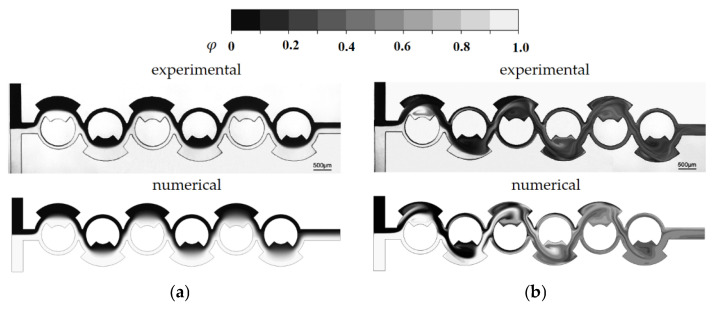
Comparison of numerical concentration contours with corresponding experimental images: (**a**) Re = 1 and (**b**) Re = 80.

Figure 4 provides a quantitative comparison between the numerical results and the corresponding experimental data by Xia et al. [44]. While there are some minor discrepancies between the two sets of data, the overall trend of the mixing index M as a function of Re is consistent. The observed discrepancy may arise from several factors, including the numerical diffusion inherent in the simulation and experimental uncertainty in empirical data. Figure 5 presents a detailed comparison between the numerical concentration contours and the experimental confocal images at Re = 1 and Re = 80. This comparison confirms that the simulated results align well with the experimental images across different Reynolds numbers. Notably, the simulated results accurately predict both the mixing pattern and the vortex flow within the mixing units at Re = 80. This agreement suggests that despite the challenges, such as numerical diffusion, the numerical approach used in this study is capable of reliably replicating the experimental observations.

## 4. Present Micromixer Based on Multiple Passages

**Figure 6:**
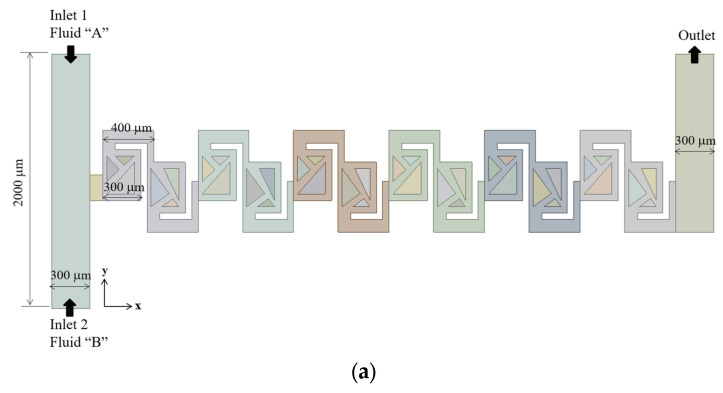

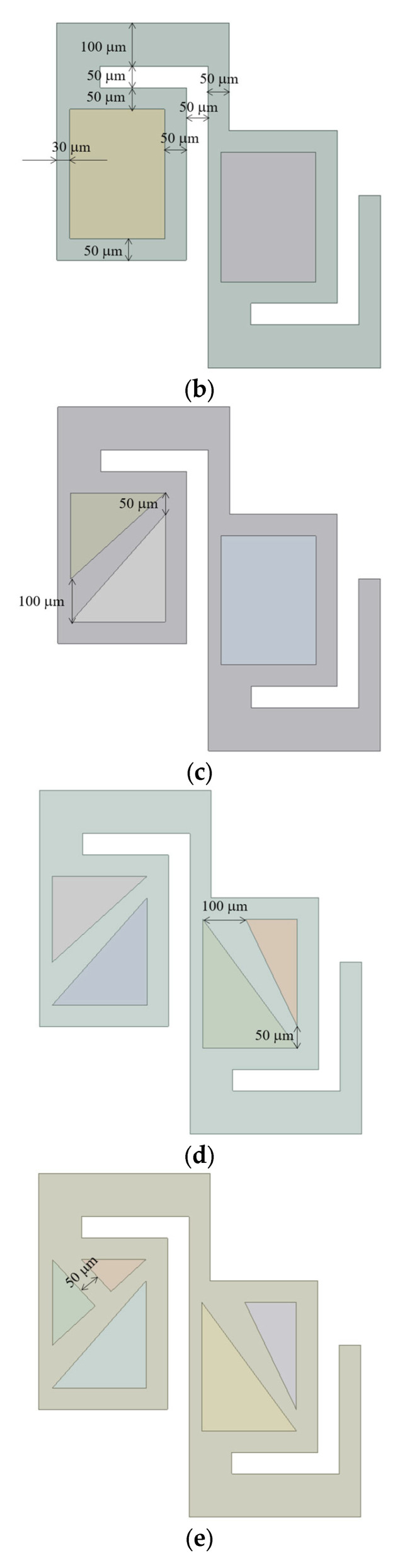

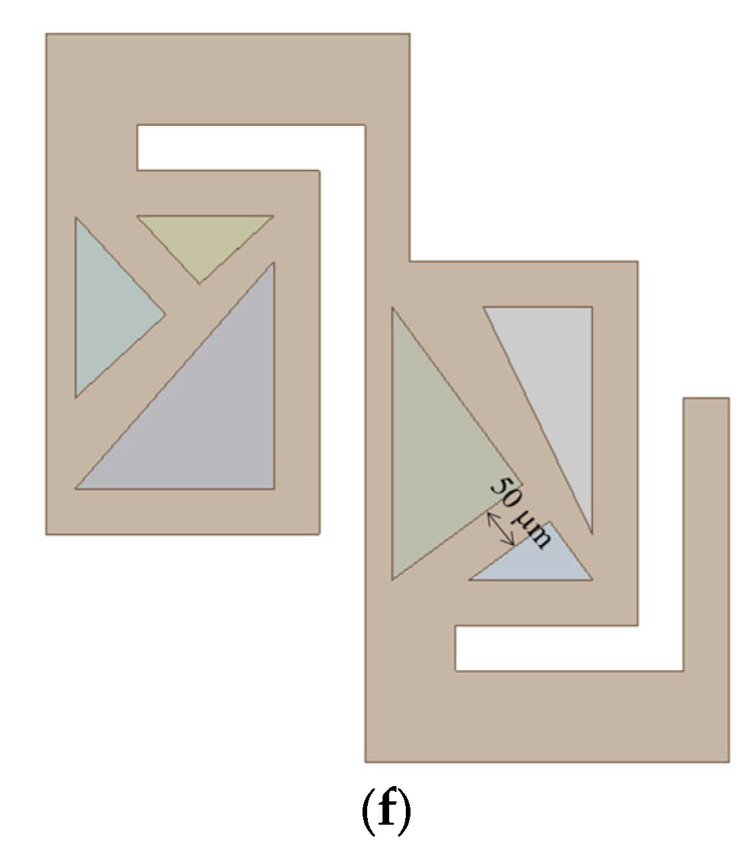
Schematic diagram of present micromixer: (**a**) front view, (**b**) baseline design, (**c**) one-passage design, (**d**) two-passage design, (**e**) three-passage design, and (**f**) four-passage design.

The micromixer under study contains six mixing units, as depicted in Figure 6a. Each of these mixing units comprises two mixing cells, which are designed with triangular structures. The triangular structures construct multiple converging passages, each characterized by its contraction ratio. The number of converging passages serves as a design parameter, influencing the mixing performance. This parameter varied from 0 to 4. Accordingly, we simulated the five different configurations to assess the effects on the mixing performance of the present micromixer. This approach allows for a systematic investigation of the relationship between the number of passages and the mixing performance. All the structures within the mixing units is 90 μm shorter than the channel height. Consequently, the multiple passages are submerged within the micromixer.

Figure 6b illustrates the baseline mixing unit, which is characterized by the absence of converging passage and includes two submerged rectangular structures. In this configuration, a portion of the flow is directed to navigate the gap between the rectangular structure and the walls of the micromixer, causing the two fluids to primarily flow over the submerged structures. In contrast, Figure 6c introduces one converging passage in the first mixing cell of each mixing unit, leading to a split-and-recombine flow pattern. This converging geometry is designed to facilitate the flow through the passage, enhancing mixing performance. Figure 6d shows a configuration with two converging passages, one located in each mixing cell. Both converging passages are constructed to follow the main flow direction, with the same contraction ratio of 0.5. Figure 6e increases the number of passages by introducing an additional bifurcation. This additional passage maintains a constant width of 50 μm, increasing the opportunities for fluids to split and recombine. Finally, Figure 6f presents the most complex geometry with another passage of constant width from that of Figure 6d.

The inlet and outlet branches have a rectangular cross-section, measuring 300 μm in width and 200 μm in depth. Each of the two inlets is 1000 μm long, while the outlet is 800 μm long. Positioned on opposite sides, the two inlets facilitate the mixing process primarily in the subsequent mixing units. The total axial length of the six mixing units is approximately 4.5 mm.

**Figure 7:**
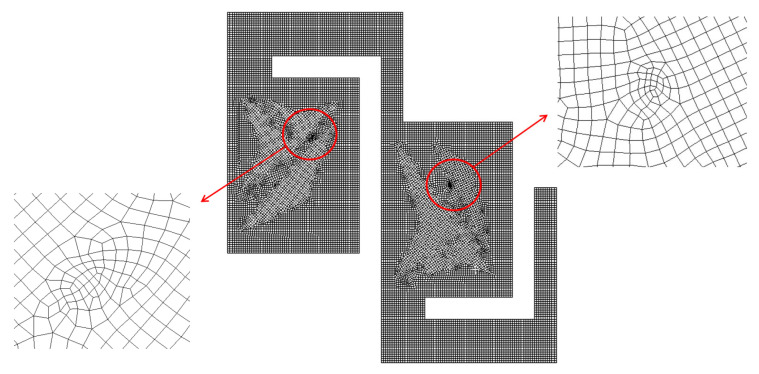
Example of mesh in a mixing unit.

The present micromixer illustrated in Figure 6a was meshed mainly with hexahedral cells. To minimize potential numerical diffusion, the cell was generated with two principles. One is to minimize unstructured cell pattern and the other is to keep equal size for all cells [45,46]. In addition, the cell size was deliberately decided through a set of preliminary simulations. These pre-simulations were carried out at Re = 0.5 for most of the designs of complicated geometry. For these simulations, the edge size of each cell was restricted smaller to a specific value. The edge size limit was varied among 4 μm, 5 μm, and 6 μm, with corresponding cell counts of 2.7 × 10^6^, 4.7 × 10^6^, and 9 × 10^6^, respectively. Figure 7 presents a detailed view of the mesh within a mixing unit. According to Okuducu et al. [47], the type of cell can largely affect numerical accuracy. Hexahedral cells are strongly recommended over triangular prism and tetrahedral cells. Consequently, hexahedral cells were mostly employed in the mesh, as illustrated in Figure 7. It has a limited number of prism cells, while tetrahedral cells were completely avoided.

The convergence index (GCI) [48,49] was used to evaluate quantitatively the uncertainty of simulation solutions. The GCI is calculated using the following formula:

(8) $$GCI=F_{s}\frac{\left|\varepsilon \right|}{r^{p}-1},$$

where *F_s_*, *p*, and *r* indicate the safety factor of the method, the order of accuracy of the numerical method, and the grid refinement ratio, respectively. ε is determined as follows:

(9) $$\varepsilon =\frac{f_{coarse}-f_{fine}}{f_{fine}},$$

where *f_coarse_* and *f_fine_* are the numerical solutions obtained with a coarse and fine grid, respectively. Based on Roache’s recommendation [48], the safety factor *F_s_* was set to 1.25. The GCI was calculated using three different edge sizes, resulting in a GCI of approximately 2.2% for an edge size of 5 μm. Reducing the edge size to 4 μm decreased the GCI to 1.6%. Consequently, 5 μm was chosen as the optimal limit, considering its favorable GCI value and a balanced trade-off between numerical accuracy and computational cost. In general, the issue of numerical diffusion becomes less significant as convective flow dominates the mixing process with an increase in the Reynolds number. However, additional validation was conducted at Re = 20, to make the choice of edge size. The GCI was 2.5% for an edge size of 5 μm. Reducing the edge size to 4 μm decreased the GCI to 1.3%.

## 5. Mixing Performance of Present Micromixer

We conducted simulations of the present micromixer across a broad range of Reynolds numbers, from 0.1 to 80. For these simulations, we assumed a uniform velocity distribution at the two inlets, with magnitude ranging from 0.21 mm/s to 0.17 m/s. This range corresponds to volume flow rates between 1.5 μL/min and 1206 μL/min. The mixing performance was assessed by calculating the degree of mixing (DOM) at the outlet, along with the associated pressure drop.

**Figure 8:**
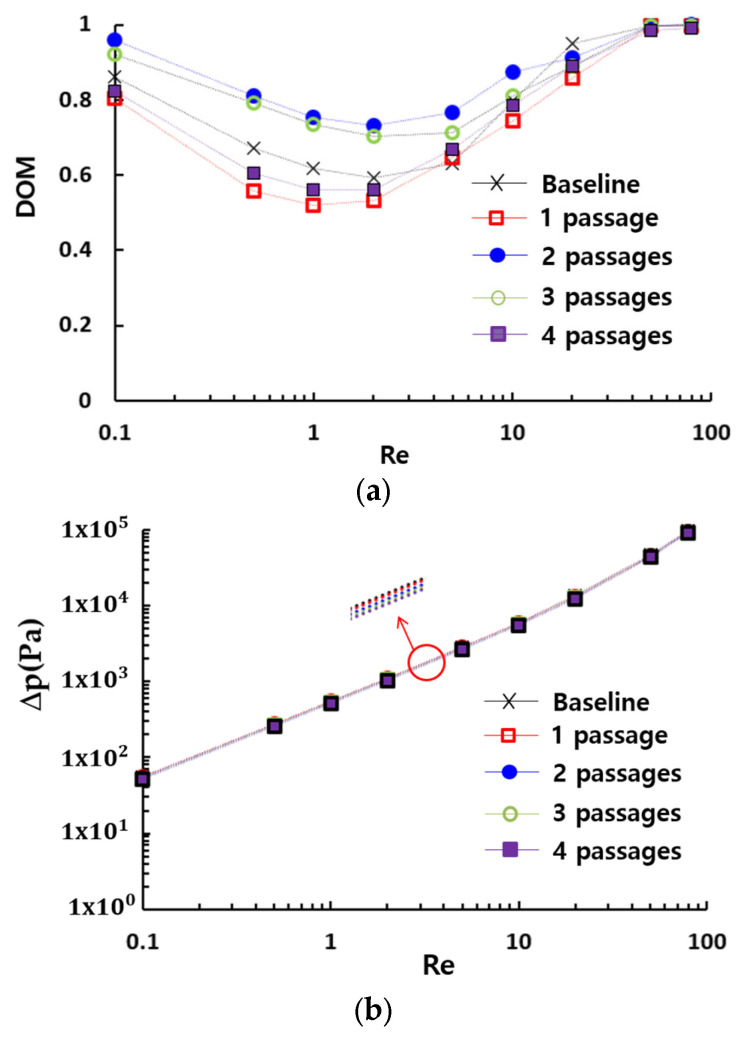

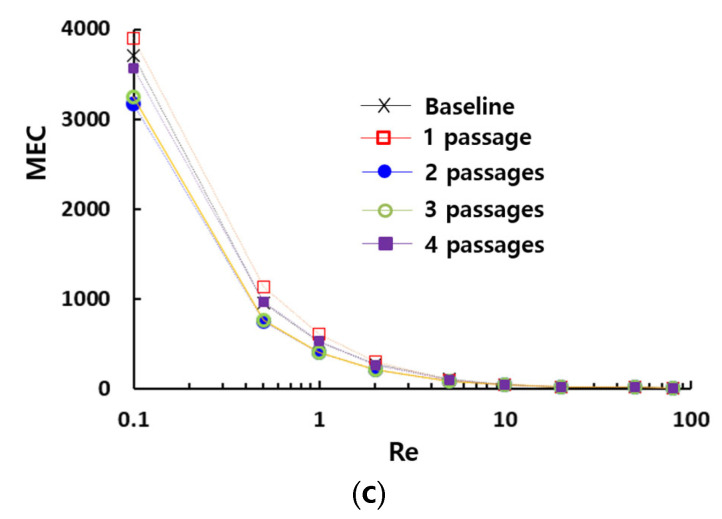
Mixing performance of the present micromixers based on geometric complexity: (**a**) DOM vs. Re, (**b**) Δπ ωσ. Pε and (**c**) MEC vs. Re.

Figure 8 presents the impact of the multiple passage design on the DOM in the present micromixer. Comparing to the DOM of the baseline design which has no additional passage, the number of passages is a crucial design parameter over a broad range of Reynolds numbers. Additionally, it can be optimized to enhance the DOM. The present micromixer, featuring a two-passage configuration, demonstrates the best mixing performance over a broad range of Reynolds numbers. Designs with fewer or more passages result in a lower DOM compared to the two-passage configuration. Meanwhile, the required pressure drop shows negligible dependence on the number of passages, as demonstrated in Figure 8b. This indicates that the number of passages can be optimized to enhance the DOM without requiring additional consideration for pressure load. This result is confirmed in Figure 8c, which compares the effectiveness of multiple passage configurations in terms of MEC. The two-passage configuration exhibits the lowest MEC, indicating that it is the most cost-effective choice.

**Figure 9:**
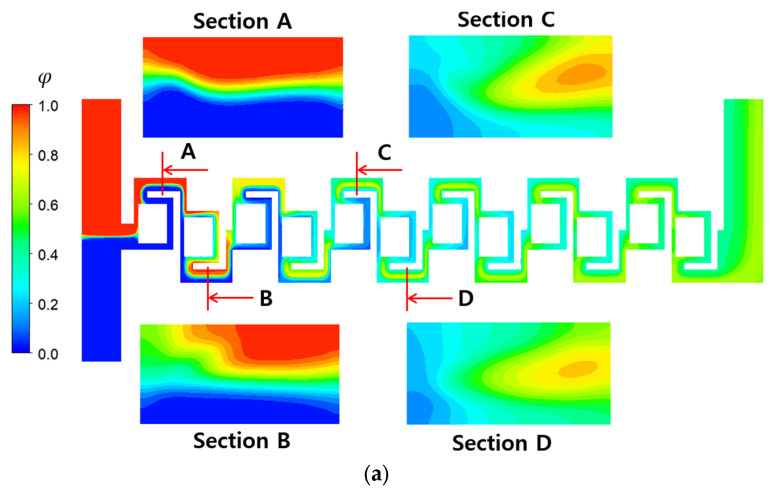

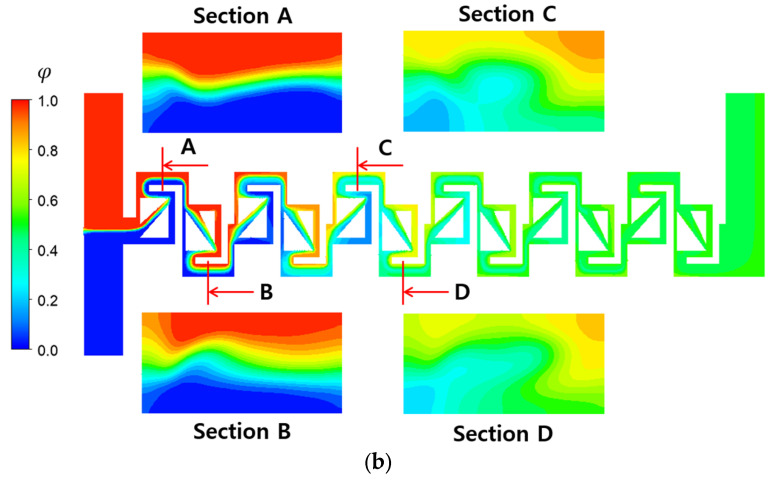
Evolution of mixing along the micromixer at Re = 0.1: (**a**) baseline and (**b**) two-passages.

Figure 9 compares the mixing evolution of the baseline and the two-passage designs, highlighting the apparent effect of the number of passages on the mixing evolution at a Reynolds number of 0.1. It illustrates the mixing progress along the mixing units on the mid-plane in the z-direction. This mixing progress is mainly achieved by molecular diffusion. The concentration of fluid A, denoted as φ, is also compared at four locations along the micromixer, illustrating how the two fluids mix on the cross-section perpendicular to the main flow direction. Differences in the φ distribution indicate the effectiveness of passage design. The two-passage design results in more dynamic mixing in the direction normal to the interface between the two fluids. This is due to the elongated interface between the two fluids, provided by the additional passage. The difference in mixing evolution between them becomes pronounced as the fluids flow downstream. Both the mixing evolution along the micromixer and the mixing on the cross-section normal to the mainstream supports that the multiple passage design is an effective concept to promote three-dimensional mixing, even at a low Reynolds number of 0.1.

**Figure 10:**
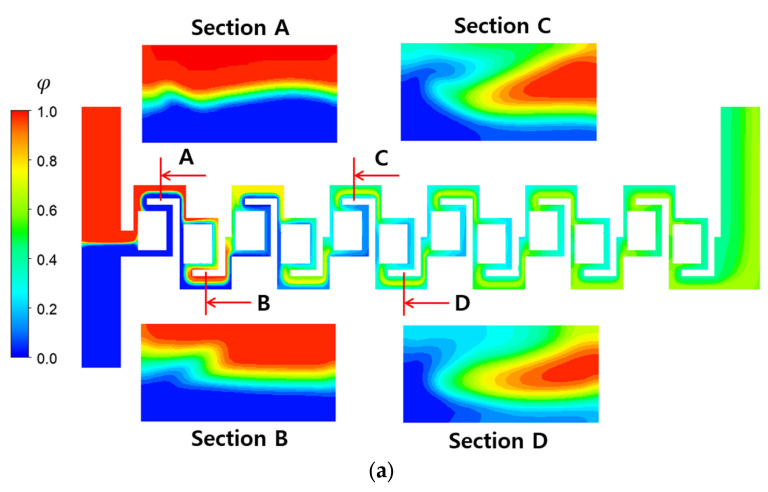

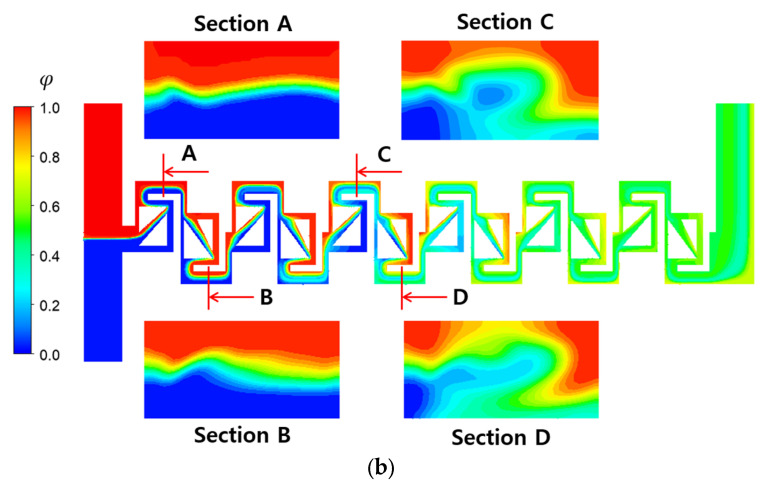
Evolution of mixing along the micromixer at Re = 1: (**a**) baseline and (**b**) two passages.

Figure 10 compares the mixing evolution of the baseline and two-passage designs at Re = 1. At this Reynolds number, mixing is influenced by both convective flow and molecular diffusion, unlike at Re = 0.1. Comparing the concentration distribution on the cross-sections A and B with those in Figure 9, it appears that the mixing in the direction perpendicular to the interface between the two fluids is quite limited. This suggests that faster fluid velocity hinders the mixing at the interface via molecular diffusion. However, mixing enhancement due to convective flow is well observed in the concentration distribution at the planes C and D. As the fluid flow downstream, the secondary flow on the cross-section perpendicular to the main stream develops further. Consequently, the two-passage design demonstrates more vigorous mixing behavior on the cross-sections C and D, compared to the baseline design.

**Figure 11:**
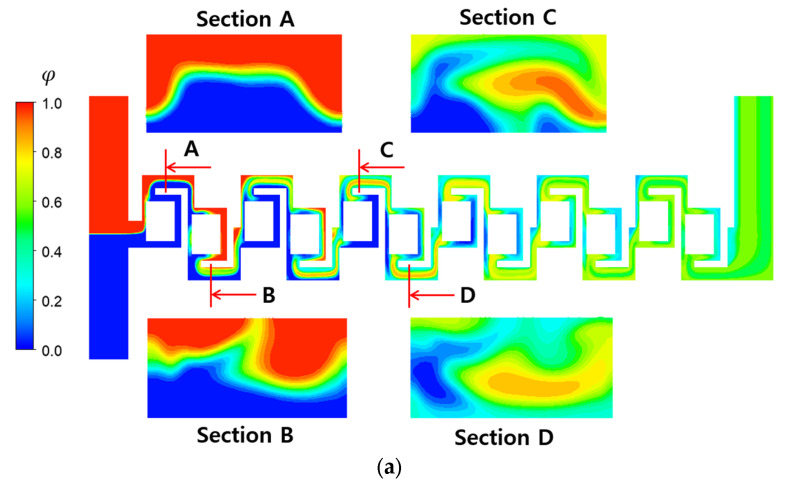

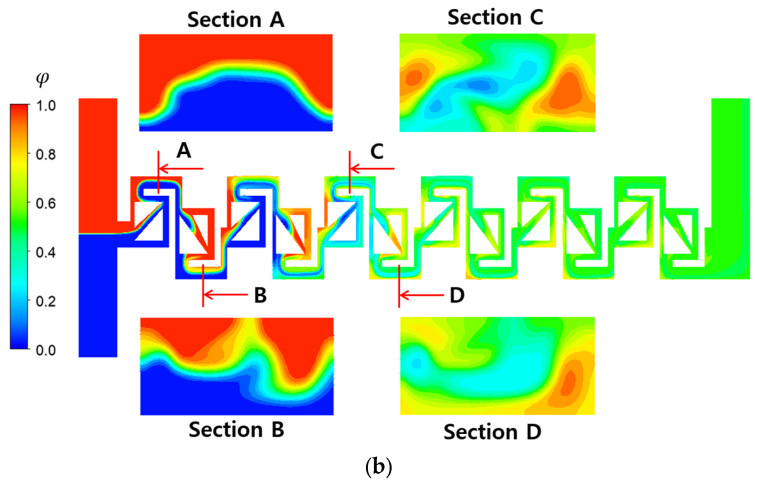
Evolution of mixing along the micromixer at Re = 10: (**a**) baseline and (**b**) two passages.

Figure 11 presents the mixing evolution along the mixing units on the mid-plane in the z- direction at a Reynolds number of 10, where the effects of convective flow on mixing becomes more pronounced. When comparing the interface of the two fluids of the two-passage design at the planes A and B, no noticeable difference is observed between them. Only the interface in the two-passages design appears a little bit curvier than in the baseline design. It suggests that convective flow dominates the mixing at this Reynolds number. The mixing due to convective flow is observed more vigorously on the cross-section as the fluids flow downstream, demonstrated at the planes C and D. This mixing enhancement is more significant at the two-passage design, compared to the baseline design. The present micromixer features multiple passages, each with varying flow velocities. Additionally, the stream undergoes several splitting and recombining processes, which increases the residence time of the contact between the two fluids. That is considered a primary mechanism for enhanced mixing across a board range of the Reynolds numbers.

**Figure 12:**
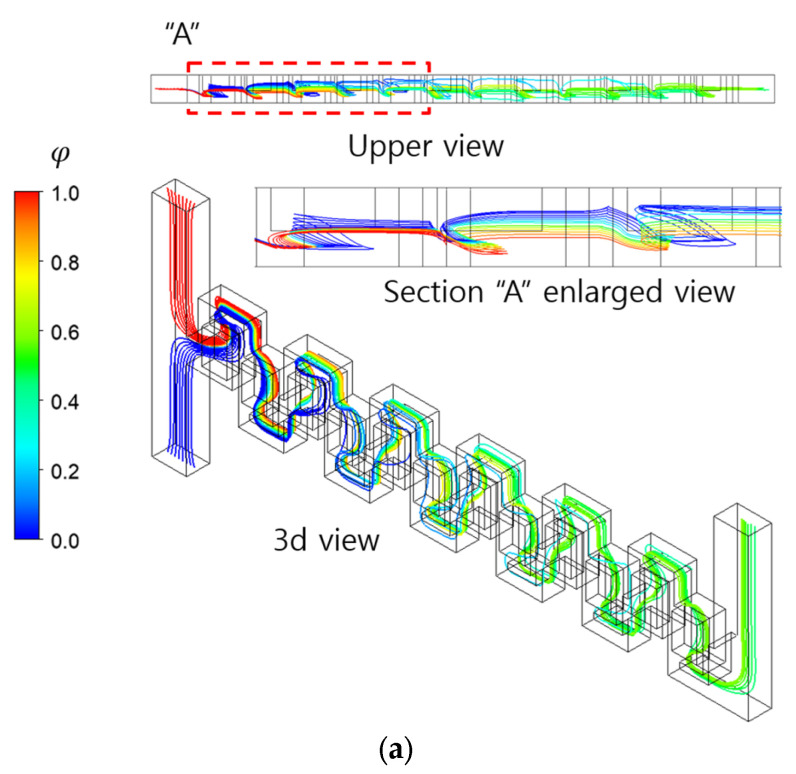

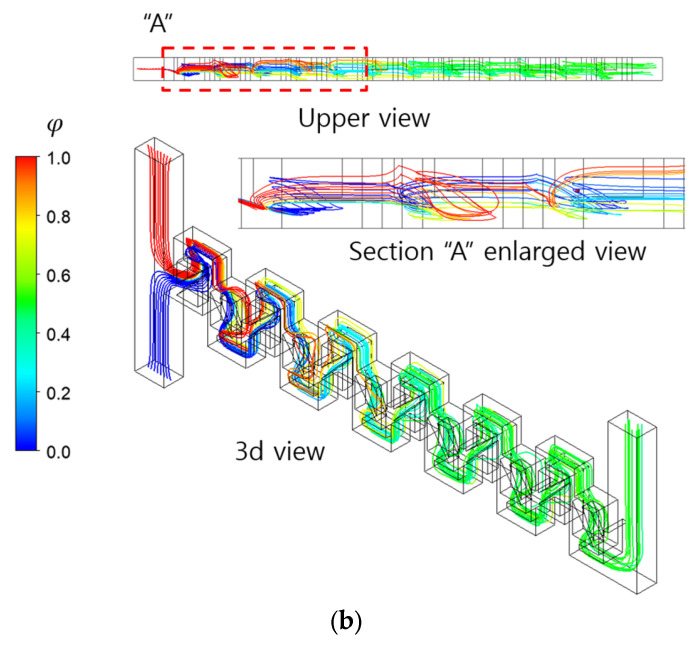
Comparison of streamline pattern along the micromixer at Re = 10: (**a**) baseline and (**b**) two passages.

Figure 12 confirms how the two-passage design enhances the mixing evolution along the micromixer at Re = 10, as observed through streamlines. The figure displays two sets of streamlines, each originating from the center line at the two inlets. In the upper view, the streamlines of the baseline design show a relatively segregated pattern, compared to those of the two-passage design. This suggests that the secondary flow in the two-passage design is more actively engaged in enhancing mixing in the cross-section, compared to the baseline design.

**Figure 13:**
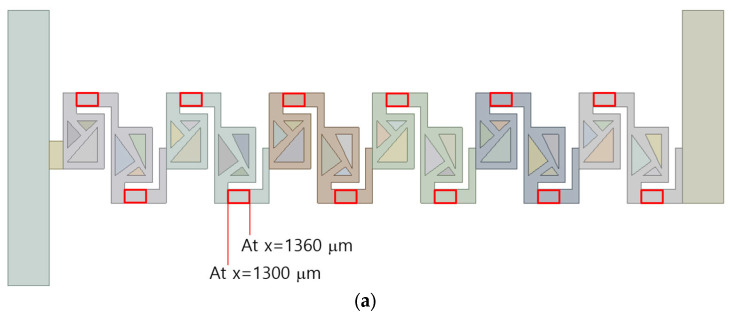

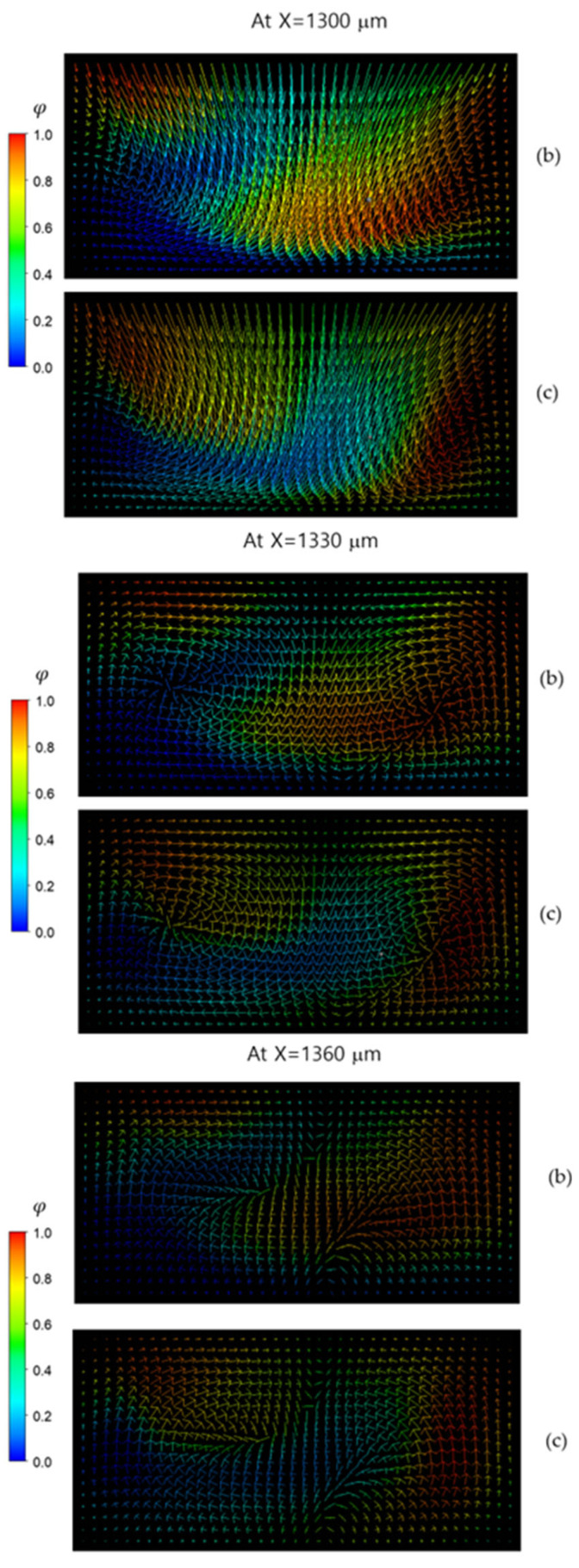
Comparison of secondary flow pattern with concentration contours at Re = 10: (**a**) secondary flow zones, (**b**) baseline and (**c**) two passages.

Figure 13 illustrates the formation of secondary flow and its impact on the mixing process at Re = 10. In each straight passage between two consecutive mixing cells, a pair of vortices is generated, developed and eventually dissipated, with the rectangular space indicated in Figure 13a. For example, (b) and (c) in Figure 13 demonstrate a pair of vortices forming at X = 1300 μm, fully developing at X = 1330 μm and beginning to dissipate at X = 1360 μm. This flow pattern is observed across all passage designs. However, in the two-passage design, fluid “A” (red in the figure) is more evenly separated by fluid “B” (blue in the figure). Therefore, mixing along the boundary of two vortices occurs more rigorously in the two-passage design.

**Figure 14:**
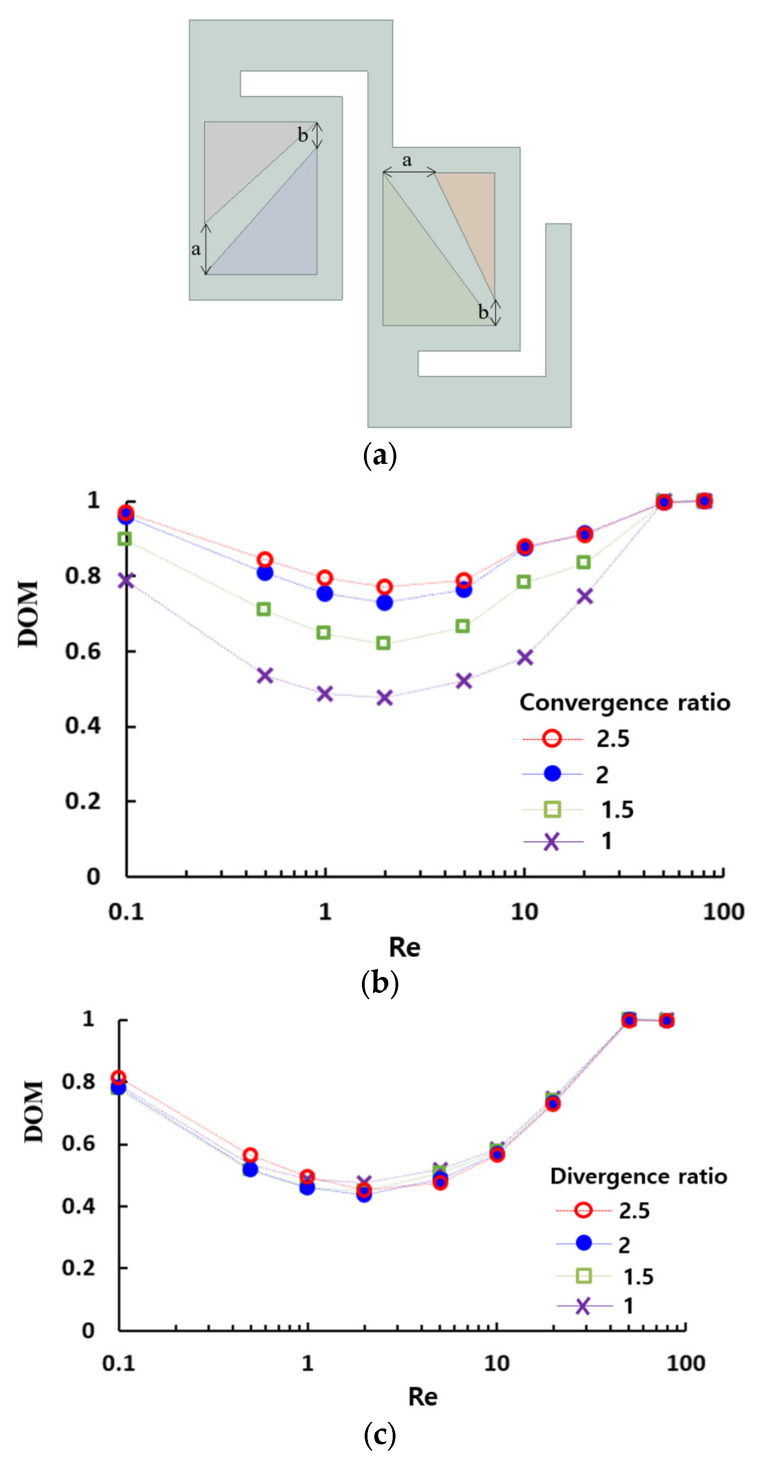
Effects of passage form factor on the mixing performance of the present micromixer: (**a**) schematic diagram, (**b**) converging passage with b = 50 μm, and (**c**) diverging passage with a = 50 μm.

We also investigated the effects of the form factor of passages on the mixing performance of the present micromixer. Figure 14a explains how the passage is defined with two geometric parameters, *a* and *b*. If *a* is larger than *b*, the passage is converging with a convergence ratio *a*/*b*. Conversely, if *a* is smaller than *b*, the passage is diverging with a divergence ratio *b*/*a*. The ratio varied from 1 to 2.5. The simulation results illustrated in Figure 14b,c demonstrate that the convergence ratio of passage has a significant impact on the mixing performance, while the divergence ratio has a negligible effect. Another finding is that the convergence ratio can be optimized to maximize the DOM over a broad range of Reynolds numbers. For the present micromixer, the optimum value of the convergence ratio is approximately 2.

**Figure 15:**
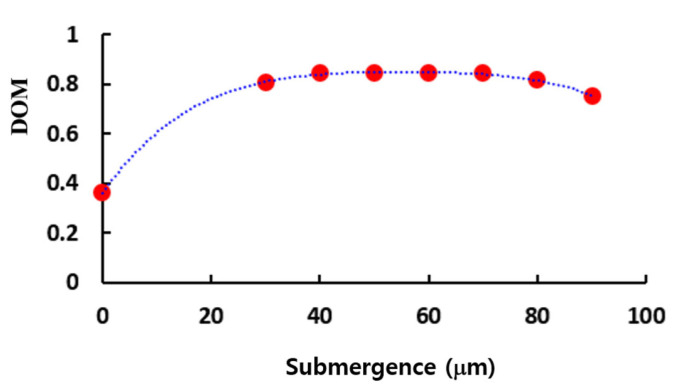
Effects of the submergence on the DOM at Re = 1.

Figure 15 presents the effects of the submergence on the DOM at Re = 1. The DOM is almost constant for a submergence in the range from 40 to 70 (μm). This is a favorable characteristic in terms of manufacturing process. The submergence of 50 (μm) shows the best DOM, so it was chosen as an optimum value.

**Figure 16:**
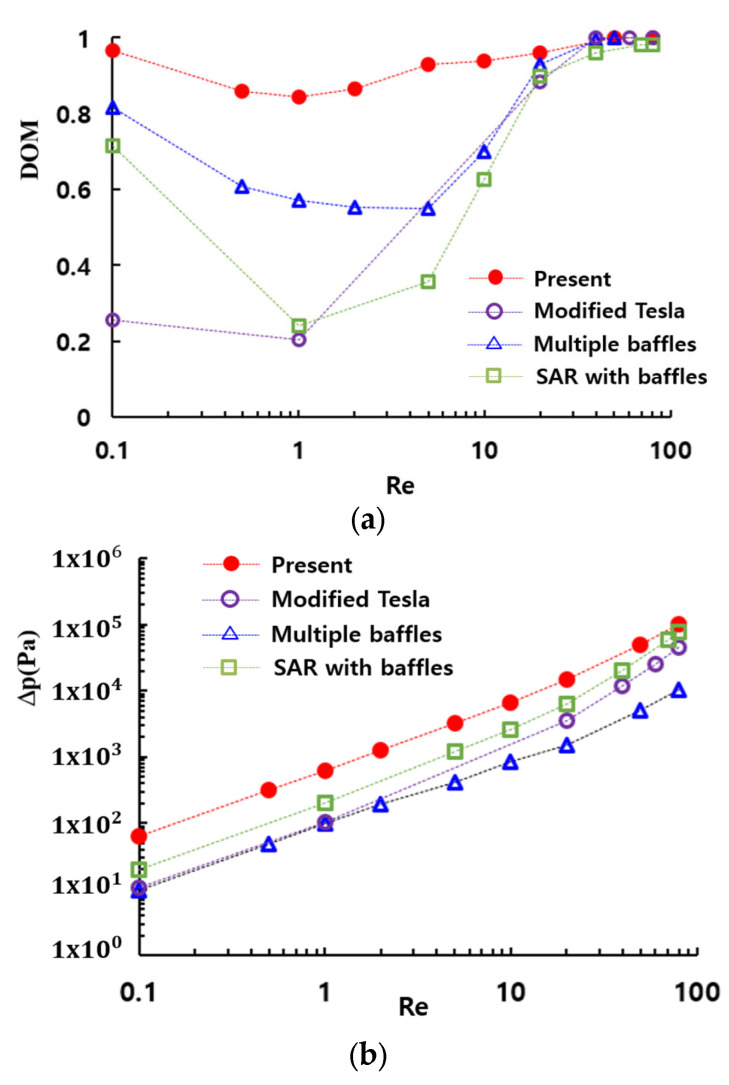
Comparison of the mixing performance of the present micromixer with other passive micromixers: (**a**) DOM vs. Re and (**b**) Δπ ωσ. Pε.

Figure 16 provides a comparative evaluation of the mixing performance of the present micromixer with several passive micromixers: a SAR micromixer with baffles [50], a micromixer with multiple baffles [30], and a modified Tesla micromixer [51]. All four passive micromixers were simulated under similar boundary conditions and physical properties. The present micromixer shows the best performance over a broad range of the Reynolds numbers. Particularly in the range of the Reynolds numbers (Re≤20), the enhancement is extraordinarily high compared to other passive micromixers. Specifically, the DOM of the present micromixer is approximately 0.75 at Re = 1, while the modified Tesla micromixer [51], the SAR micromixer with baffles [46], and the passive micromixer with multiple baffles [30] achieve DOMs of 0.2, 0.49, and 0.57, respectively. This corresponds to 32% improvement over the passive micromixer with multiple baffles [30]. This result suggests that the present micromixer based on multiple passages could serve as a promising design for enhancing mixing performance in the molecular diffusion and transition regime. However, it should be noted that this enhancement is accompanied by an increase in the required pressure drop, as depicted in Figure 16b.

## 6. Conclusions

In this paper, we introduced a novel passive micromixer based on multiple passages and thoroughly analyzed its mixing performance. The present micromixer consists of six mixing units, each housing two mixing cells containing multiple passages. Therefore, the number of passages and their aspect ratios are key design parameters. To ascertain the significance of this design, we simulated five distinct designs varying the number of passages from 0 to 4. Two different types of passages, converging and diverging, were investigated, with the aspect ratio varying from 1 to 2.5.

Simulation results demonstrate that the number of passages significantly impacts the mixing performance. The two-passage design exhibits the best performance over a broad range of the Reynolds numbers. Converging passages result in better performance compared to diverging passages. Additionally, the convergence ratio has a significant impact on the mixing performance, with the optimal convergence ratio for the present micromixer being approximately two. The DOM remained nearly constant when the multiple passages were submerged in the range of 40 (μm) to 70 (μm), which is advantageous for manufacturing process.

The present micromixer demonstrates a significant enhancement in the DOM within the low and intermediate range of Reynolds numbers (Re≤20) compared to other passive micromixers, such as a modified Tesla micromixer, a passive micromixer with multiple baffles and a SAR micromixer with baffles. Moreover, the DOM of the present micromixer is larger than 0.84 across the entire range of Reynolds numbers simulated in this paper. These results indicate that the present micromixer based on multiple passages could be a potential design for achieving high mixing performance throughout a wide range of Reynolds numbers. In addition, the geometry is quite simple, even compared with other planar micromixers.

The mixing enhancement in the molecular diffusion dominance regime of mixing is primarily attributed to the elongated interface between the two fluids, resulting in a wavier and broad pattern. On the contrary, the mixing enhancement in the transition regime of mixing is due to a secondary flow generated on the cross-section normal to the main stream direction. The intensity of this secondary flow is noticeably influenced by the number of multiple passages constructed within each mixing cell. The optimal number of flow passages for the present micromixer design was found to be two.

## Data Availability

The original contributions presented in this study are included in the article. Further inquiries can be directed to the corresponding author.
